# Genotype clinical phenotype analysis of 35 cases of hereditary spherocytosis in children

**DOI:** 10.3389/fped.2025.1650295

**Published:** 2025-08-20

**Authors:** Huang Duowen, Guo Xia, Gao Ju

**Affiliations:** ^1^ Department of Pediatrics, West China Second Hospital, Sichuan University, Chengdu, China; ^2^ Key Laboratory of Birth Defects and Related Diseases of Women and Children, Ministry of Education, Sichuan University, Chengdu, China

**Keywords:** pathogenic genes, genotype and phenotype, red blood cell structure, hereditary spherocytosis, whole exome gene sequencing

## Abstract

**Objective:**

Hereditary spherocytosis (HS) is a common red blood cell membrane disease. It is currently clear that mutations in genes such as ANK1, SPTB, SPTA1, SLC4A1, EPB4.2 can cause the loss of their corresponding encoded proteins. However, there is a lack of reports in China on the association analysis between HS genotypes and clinical phenotypes, aiming to reveal whether there are differences in the corresponding clinical phenotypes of the same disease when genotypes are different.

**Methods:**

35 children with HS who underwent complete whole exome gene sequencing in the Department of Pediatric Hematology at West China Second Hospital of Sichuan University from February 2014 to February 2024. Grouping according to different mutated genes/mutation types, and statistical analysis of blood routine and liver function indicators between different groups; Mann Whitney test analysis was used for inter group data processing, and significant differences were considered when both sides were *p* < 0.05.

**Results:**

Compared with the SPTB group, the ANK1 group had significantly lower RBC (*p* = 0.021) and HGB (*p* < 0.01), but the differences in other indicators were not statistically significant (*p* > 0.05).

**Conclusions:**

After excluding potential influencing factors such as splenectomy, the anemia symptoms in ANK1-HS patients were more severe than those in SPTB-HS patients. However, there was no statistically significant difference in indicators between HS patients with different types of gene mutations.

## 1 Introduction

Hereditary spherocytosis (HS) is a common type of hereditary hemolytic anemia, with primary clinical manifestations including hemolytic anemia, jaundice, and splenomegaly. It is currently believed that the disease is primarily genetically related, with genetic polymorphisms potentially involved in the pathogenesis. HS is a disease reported worldwide and is the most common type of hereditary anemia in populations of Nordic descent, with a prevalence of approximately 1 in 2,000 in Nordic populations. There are currently no definitive diagnostic criteria for the disease. The clinical severity of HS varies among patients, with some presenting with mild anemia and jaundice, while others have no clinical manifestations. However, some patients exhibit severe hemolytic anemia and require regular blood transfusions. Among these genes, five genes are associated with abnormalities in the encoding of red blood cell membrane skeleton proteins: the ANK1 gene encodes the anchor protein, the SPTB gene encodes the β-hemoglobin protein, SPTA encodes α-spectrin, SLC4A1 encodes band 3 protein, and EPB42 encodes protein 4.2]. These gene mutations result in abnormal membrane anchoring on the red blood cell surface, altering the normal biconcave disc shape of red blood cells and causing spherocytosis. Spherical red blood cells have poor deformability and are easily destroyed when passing through the splenic sinuses, leading to hemolytic anemia. Due to increased red blood cell destruction, the bone marrow undergoes stimulatory hematopoiesis as a compensatory mechanism. At this stage, bone marrow smears often show active erythroid proliferation, while peripheral blood may exhibit increased reticulocyte counts, hyperbilirubinemia, and imaging studies may indicate gallstones and splenomegaly.

This study aims to further clarify the consistency between HS gene diagnosis and clinical diagnosis, explore the feasibility of routinely applying gene diagnosis to HS diagnosis, and analyze the characteristics of HS-related gene mutations in southwestern China.

## 2 Data and methods

### 2.1 General information

#### 2.1.1 Source of cases

The cases were collected from February 2014 to February 2024 at the Department of Pediatric Hematology and Oncology, West China Second University Hospital, Sichuan University. The cases involved children with hemolytic anemia who underwent whole-exome gene testing and were diagnosed with HS. With the informed consent of the children and their guardians, peripheral blood samples were collected from the children and their parents. The gene testing was performed by Kangsheng Global. The sequencing depth of this test was ≥100×, with target region coverage ≥95%. For *de novo* mutations, the Human Gene Mutation Database (HGMD) and the Online Mendelian Inheritance in Man (OMIM) database were used to identify new mutation sites. Pathogenicity analysis was conducted according to the sequencing variant interpretation guidelines established by the American College of Medical Genetics and Genomics (ACMG) (1).

#### 2.1.2 Diagnostic process (2, 3)

The diagnosis of HS in the past was mainly based on clinical manifestations and related family history. The HS diagnostic protocol developed by Shen proposed that if a patient has more microspherocytes (>10%) in the peripheral blood, with increased red blood cell permeability and fragility, and a positive family history, he/she, regardless of the symptoms, can be diagnosed with HS. The HS diagnostic guidelines developed by Bolton-Maggs (4) proposed that HS can be confirmed with no need to perform other tests when patients have a positive family history, typical clinical features, and abnormal laboratory test results (peripheral blood spherocytosis, MCHC increase, reticulocyte increase). However, the existing HS diagnosis protocols are usually difficult to be used by the clinicians in the following aspects:

(1) When the patients' parents have trait and mild HS with no obvious symptoms, it is easy to report this as a negative family history.
(2) The laboratory tests, such as MCHC, OFT, and peripheral red blood cell morphology examinations, are not sufficiently sensitive or specific.
(3) Glucose-6-phosphate dehydrogenase (G6PD) deficiency, AIHA, and other diseases can be accompanied by peripheral blood spherocytes (5).
(4) OFT can be positive for hereditary elliptocytosis (HE), AIHA, and other diseases (4).
(5) The accuracy of the peripheral red blood cell morphology examination is related to the time of specimen placement and the level of inspectors.

**Figure 1:**
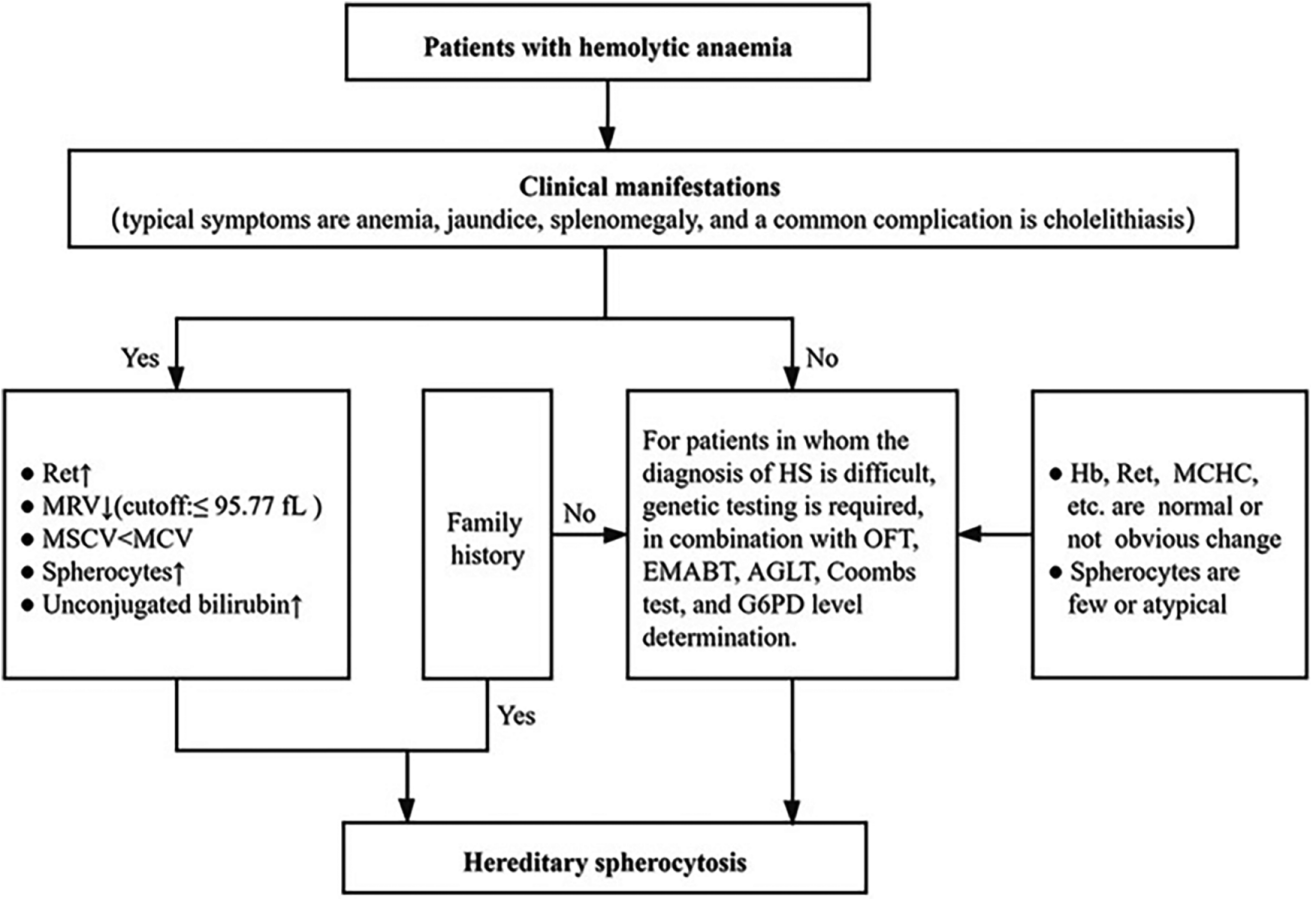
Diagnostic process of HS (3).

In view of the high incidence rate of HS and the difficulty of HS diagnosis, the Center has adopted a simple and practical HS diagnosis scheme [Figure 1 (3)]:

A. Clinical manifestations: typical symptoms are anemia, jaundice, splenomegaly, and a common complication is cholelithiasis.
B. Routine laboratory tests: Hb level is normal or decreased; Ret is normal or increased; MCHC is normal or increased; MRV is decreased; MSCV < MCV; spherocytes may be increased in number; and serum total bilirubin is increased, mainly presenting with an increase in the level of unconjugated bilirubin.
C. Family investigation: most patients present with autosomal dominant inheritance and have the same examination results and clinical manifestations as one of their parents or other family members.
D. Genetic testing and other screening tests: for patients in whom the diagnosis of HS is difficult, genetic testing is required, in combination with OFT, EMABT, AGLT, Coombs test, and G6PD level determination.

#### 2.1.3 Diagnostic criteria for anemia

According to the recommendations of the China Pediatric Blood conference: Anemia Diagnosis: in the neonatal period hb < 145 g/L; from 1 month old to 4 months old Hb < 90 g/L; from 4 month old to 6 months old Hb < 100 g/L; after 6 months of age Hb < 110 g/L.

#### 2.1.4 The application of whole-exome gene sequencing

In the diagnosis of hereditary spherocytosis, genetic testing is not a first-line method, but genetic diagnosis should be considered at the following specific times:

First: Atypical clinical manifestations or laboratory results
  - Suspected of HS but with ambiguous or negative results from routine tests such as OFT and EMA.
  - Mild or subclinical cases (such as mild hemolysis or no typical spherocytosis).
Second: When differential diagnosis is difficult
  - When it is difficult to distinguish from other hereditary hemolytic anemia (such as hereditary ellipticosis, membrane protein disease)
  - Exclude atypical hemolytic diseases (such as congenital anemia with abnormal erythropoiesis, CDA)
Third: Guide treatment decisions
  - Before planning splenectomy, determine the genotype to assess the risk of postoperative thrombosis and other related factors.
  - Assess the severity of the disease and the risk of long-term complications.

#### 2.1.5 Methods and statistics

Clinical data from 35 pediatric patients diagnosed with HS through genetic testing were collected. The enrolled patients were grouped according to mutation gene type and mutation pattern, and statistical analysis was performed on multiple laboratory test indicators, including blood routine and liver function. Since the aim was to compare the differences between the medians of two independent samples and the data did not meet the requirements of normal distribution, the Mann–Whitney *U* test was used for intergroup data analysis. A difference was considered statistically significant when the two-sided *p*-value was <0.05.

## 3 Result

### 3.1 General condition of the patient

A total of 35 children diagnosed with hereditary spherocytosis through genetic testing were included in this study. None of the children were related by blood, with a male-to-female ratio of 16:19 (close to 1:1). The median age at presentation was 2 years and 4 months.

### 3.2 Basic information and clinical manifestations of 35 pediatrics patients with HS

In this study, a total of 35 pediatric patients were enrolled. There were no familial relationships among the enrolled patients, and all presented with varying degrees of anemia symptoms at the time of presentation, including 16 males and 19 females, with a male-to-female ratio of approximately 1:1 (16:19); the median age at presentation was 2 years and 4 months (ranging from the neonatal period to 12 years). Among these HS patients, some received blood transfusion therapy after admission, while others were treated with antibiotics for infection. None of the enrolled patients had undergone splenectomy or blood transfusion therapy at the time of enrollment (Table 1).

**Table 1:** The basic clinical information of 35 children with HS.

Characteristic	Total cohort (*n* = 35)
Sex	
Male	16 (46%)
Female	19 (54%)
Age	
Median (25%–75% ile)	2 (Neonatal period-12)
Haemoglobin level (g/L)	
Median (25%–75%ile)	72.00 (53–138)
Absolute reticulocyte count (×10^9^/L)	
Median (25%–75% ile)	0.3087 (0.0082–0.6621)
Transfusion (*n*, %)	
(≥1 red cell transfusion)	34 (97%)
Genetic aetiology for HS (*n*, %)	
ANK1 (ankyrin)	19 (54%)
SPTB (β spectrin)	9 (26%)
SLC4A1 (Band-3)	3 (9%)
SPTA (α spectrin)	4 (11%)

### 3.3 Laboratory tests

All test results were obtained prior to splenectomy and blood transfusion therapy. Among the 35 children with HS, 16 (45.71%) were male and 19 (54.29%) were female. RBC was 2.745 (1.98–4.62) × 10^12^/L, Hb was 72.00 (53–138) g/L, PLT was 294.0 (30–593) × 10^9^/L, HCT was 23.25 (15.8–42.3) %, MCV was 81.55 (62.8–91.2) fL, MCH was 27.05 (17.4–31.9) pg, MCHC was 336.00 (267–373) g/L, RDW-SD was 61.15 (38.8–76.8) fL, RDW-CV was 21.95 (14.3–33.2)%, RET# was 0.3087 (0.0082–0.6621) × 10^9^/L, RET was 9.755 (0.36–19.62)%, TBIL was 43.30 (8.6–634.9) μmol/L, DBIL was 7.20 (0.00–374.50) μmol/L, and IDBL was 30.45 (4.5–260.4) μmol/L. Splenomegaly or gallstones were present in 19 cases (54.28%).

Among these, 19 cases (54.29%, 19/35) had ANK1 gene mutations, and 9 cases (25.71%, 9/35) had SPTB mutations. Among the 35 pathogenic genes associated with HS, all mutation sites showed differences. Among these, 17 mutations were *de novo*, 10 were maternally derived, 7 were paternally derived, and 1 was a compound heterozygous mutation jointly produced by both parents. Among these 35 pathogenic mutations, there were 16 nonsense mutations, 8 frameshift mutations, and 2 splicing mutations, with 27 of these mutations being reported for the first time.Pathogenicity assessment was performed according to ACMG guidelines, and all were pathogenic or likely pathogenic gene mutations (Tables 2, 3).

**Table 2:** Genetic mutation results related to HS children.

Number	Pathogenicity genes	Mutated chromosomal loci	Nucleic acid changes (exon number)	Amino acid changes (variant number)
1	ANK1	Chr8:41591518	c.199 (exon3) A>T	p.K67X
2	ANK1	Chr8:41559101	c.2428 (exon22) G>C	P.V810l
3	ANK1	Chr8:41582033	C.651_652 (exon7) del	p.E217fs
4	ANK1	Chr8:41591512	C.205delA (exon3)	P.169fs
5	ANK1	Chr8:41553939	C.2902 (exon26) C>T	p.E968X
6	ANK1	Chr8:41585425	C.426+1 (exon5) G>T	–
7	ANK1	Chr8:41553976	C.2861_2865 (exon26) del	P.V954fs
8	ANK1	Chr8:41554038	C.2803 (exon26) C>T	P.R935X
9	ANK1	Chr8:41591515	C.202 (exon3) G>T	P.E68X
10	ANK1	Chr8:41566388	C.1905 (exon17) dupT	P.V636fs
11	ANK1	Chr8:41552844	C.2966 (exon27) T>A	P.V989E
12	ANK1	Chr8:41553942	C.2899 (exon26) G>T	P.E967X
13	ANK1	Chr8:41552280	C.3157 (exon28) C>T	P.R1053X
14	ANK1	Chr8:41551575	C.3373 (exon29) C>T	P.Q1125X
15	ANK1	Chr8:41529924	C.5044 (exon38) C>T	P.R1682X
16	ANK1	Chr8:41584836	C.358 (exon5) C>T	P.Q120X
17	ANK1	Chr8:41575701	C.1128_1129 (exon11) InsCTTACAC	P.I377fs
18	ANK1	Chr8:41519398	C.5540 (exon41) delA	P.E1847fs
19	ANK1	Chr8:41575734	C.426+1 (exon5) G>T	–
20	BRCA2	Chr13:32971071	C.9538 (exon26) C>T	P.L3180F
21	BRCA2	Chr13:32911937	C.3445 (exon11) A>G	P.M1149V
22	EPB41	Chr1:29362384	C.1307 (exon9) G>A	P.R436Q
23	SLC4A1	Chr17:42330519	C.2278 (exon17) C>T	P.R760W
24	SLC4A1	Chr17:42330695	c.G2102Aexon17	p.G701D
		Chr17:42337179	c.C607Texon7	p.Q 203X
25	SLC4A1	Chr17:42330519	C.2278 (exon17) C>T	P.R760w
26	SPTA1	Chr1:158587845	C.6532 (exon46) G>A	P.A2178T
27	SPTB	Chr14:65271706	c. (exon2) 215A>T	P.D84V
28	SPTB	Chr14:65245883	c.4555 (exon21) A>C	p.K1519Q
29	SPTB	chr14:65240123	c.4993 (e xon24) C>T	p.Q1665X
30	SPTB	chr14:65239630	c.5220_5221del (exon25)	p.G1740fs
31	SPTB	chr14:65270357	c.442 (exon3) G>T	p.G148C
32	SPTB	chr14:65249058	c.4216 (exon19:) G>A	p.D1406N
33	SPTB	chr14:65220312	c.6544_6545 (exon32) insT	p.E2182fs
34	SPTB	Chr14:65268524	C.594 (exon5) dupT	p.T199fs
35	SPTB	Chr14:64775238_64775251	C.4716_4729del	p.G1575fs

**Table 3:** Pathogenicity assessment of gene mutations in children with HS.

Number	Genetic variant	ACMG classification of pathogenicity levels	Pathogenic evidence
1	ANK1 c.199A>T	Pathogenic	PSV1 + PM2 + PP1
2	ANK1 c.2428G>C	Pathogenic	PSV1 + PM2 + PP1 + PP4
3	ANK1 c.651_c.652del	Pathogenic	PSV1 + PM2 + PM5
4	ANK1 c.205delA	Pathogenic	PSV1 + PM2 + PP1
5	ANK1 c.2902C>T	Pathogenic	PSV1 + PM2 + PP1
6	ANK1 c.426+1G>T	Pathogenic	PVS1 + PS2
7	ANK1 c.2861_2865del	Likely pathogenic	PS2 + PM2 + PM6
8	ANK1 c.2803C>T	Pathogenic	PVS1 + PS2 + PM2
9	ANK1 c.202G>T	Pathogenic	PVS1 + PS2 + PM2
10	ANK1 c.1905dupT	Pathogenic	PVS1 + PS2 + PM2
11	ANK1 c.2966T>A	Pathogenic	PVS1 + PS2 + PM2
12	ANK1 c.2899G>T	Pathogenic	PVS1 + PS2 + PM2
13	ANK1 c.3157C>T	Pathogenic	PVS1 + PS1 + PS2
14	ANK1 c.3373C>T	Pathogenic	PVS1 + PS2 + PM2
15	ANK1 c.5044C>T	Pathogenic	PVS1 + PS2 + PM2
16	ANK1 c.358C>T	Pathogenic	PVS1 + PS2 + PM2
17	ANK1 c.1128_1129InsCTTACAC	Pathogenic	PVS1 + PS2 + PM2
18	ANK1 c.5540delA	Pathogenic	PVS1 + PS2 + PM2
19	ANK1 c.426+1 (exon5) G>T	Pathogenic	PVS1 + PS1 + PS2
20	BRCA2 c.9538C>T	Pathogenic	PVS1 + PS1
21	BRCA2 c.3445A>G	Likely pathogenic	PVS1 + PM1
22	EPB41 c.1307G>A	Likely pathogenic	PM2 + PM5 + PM6
23	SLC4A1 c.2278C>T	Likely pathogenic	PS4 + PM1 + PM5 + PP3
24	SLC4A1 c.2102G>A c.607C>T	Pathogenic	PVS1 + PS1
25	SLC4A1 c.2278C>T	Pathogenic	PVS1 + PS1
26	SPTA1 c.6532G>A	Likely pathogenic	PM2 + PM5 + PM6
27	SPTB c.215A>T	Likely pathogenic	PM2 + PM5 + PM6
28	SPTB c.4555A>C	Likely pathogenic	PM2 + PM5 + PM6
29	SPTB c.4993C>T	Pathogenic	PVS1 + PS2 + PM2
30	SPTB c.5220_5221del	Pathogenic	PVS1 + PS2 + PM2
31	SPTB c.442G>T	Likely pathogenic	PS2 + PM2
32	SPTB c.4216G>A	Likely pathogenic	PS2 + PM2
33	SPTB c.6544_ 6545insT	Pathogenic	PVS1 + PS2 + PM2
34	SPTB c.594dupT	Pathogenic	PVS1 + PM2 + PP1
35	SPTB c.4716_4729del	Likely pathogenic	PS2 + PM2

## 4 Association analysis between different HS genotypes and clinical phenotypes

**Figure 2:**
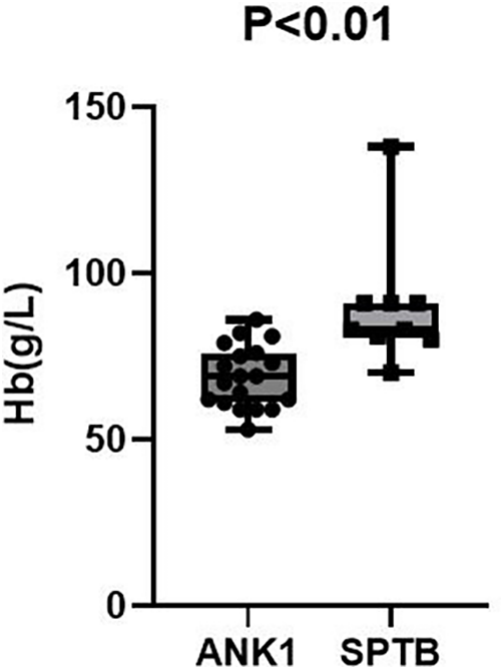
Hb *P* < 0.01.

**Figure 3:**
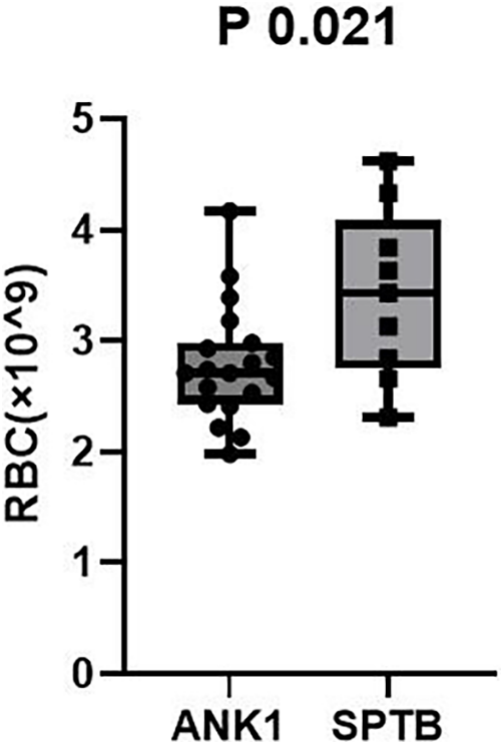
RBC *P* = 0.021.

Among 35 pediatric patients with hereditary spherocytosis, the patients were grouped based on two different gene mutation types, ANK1-HS and SPTB-HS, and intergroup comparisons were conducted to compare blood routine and liver function indicators between the two groups. It was found that children with ANK1 mutations had significantly lower RBC (*p* = 0.021) and HGB (*p* < 0.01) levels compared to those with SPTB gene mutations, but there were no statistically significant differences between the two groups in other indicators, suggesting that children with ANK1-HS have more severe anemia than those with SPTB-HS (Figures 2, 3).

## 5 Comparison of clinical characteristics of different mutation types of HS

To further clarify whether different types of gene mutations affect the clinical phenotype of HS, 35 children with hereditary spherocytosis were grouped based on nonsense mutations and frameshift mutations. Blood counts and liver function indicators were compared between the two groups, and no significant statistical differences were found between the two groups (*p* > 0.05, HGB Cohen *d* = 0.67). This suggests that different types of gene mutations do not affect the severity of clinical symptoms in children with HS.

## 6 Discussion

HS exhibits significant variability in clinical phenotypes and genotypes. The incidence of HS varies across different regions and ethnic groups. This highlights the significant role of biological factors and genetic background in the onset and progression of HS. The mutation profiles of disease-associated genes also differ across regions. There are no so-called hotspot mutations in the gene mutations in HS. Most of the mutations are sporadic and unique to individual patients or their families (6).For example, in Brazil, the prevalence of SPTB mutations is 34.6%, making it the key variant gene for HS in Brazil (7). The mutation rate of ANK1 ranges from 40%–65% in the United States and Europe, but only 5%–10% in Japan. In a cohort of 95 Dutch HS patients, the positive rate of HS genetic testing was 89% (85/95). In the ranking of pathogenic genes, the top three were SPTA1 (36%, 31/85), ANK1 (27%, 23/85) and SPTB (20%, 17/85) (8).In a cohort of 166 HS patients from Canada, the positive detection rate reached 97% (160/166), and the top three pathogenic genes were ANK1 (49%, 79/160), SPTB (33%, 52/160) and SLC4A1 (13%, 21/166) (9).These findings suggest genetic differences exist among different countries and ethnic groups.

In this study (with cases primarily from southwestern China), 19 cases (54.29%) had ANK1 mutations, and 9 cases (25.71%) had SPTB mutations. To date, reports of HS cases caused by EPB42 gene mutations leading to defects in the encoded 4.2 protein are extremely rare, whether in adult or pediatric settings (10, 11). Additionally, a review (12) of all relevant literature published between 2000 and 2020 on Chinese HS patients, which covered genetic findings and clinical data, listed a total of 158 variants, with pathogenic gene mutation frequencies as follows: ANK1 (46%), SPTB (42%), SLC4A1 (11%), and SPTA1 (1%). This finding revealed differences in the distribution patterns of HS gene mutations across regions and further indicated that ANK1 and SPTB are the key genes driving the onset of HS in Chinese patients.

Among the 35 children with HS included in this study, 16 had nonsense mutations (45.71%), 8 had frameshift mutations (22.86%), 7 had missense mutations (20.00%), and 2 had splicing mutations (5.71%), among others. This suggests that the primary mutation types in the HS gene include nonsense mutations, frameshift mutations, and missense mutations, which is consistent with most reported findings (11, 13, 14). However, in a study of 28 HS patients in Hubei Province, missense mutations were the most common mutation type (9/28) (14), while in Wang D's (12) study, missense mutations were relatively rare. This phenomenon may be related to geographical differences, and the current studies have limited sample sizes. This conclusion requires further multi-center, large-scale sample studies for confirmation.

Currently, there is no consensus on the relationship between different types of gene mutations and their phenotypes. Studies by Aggarwal (15) and Tole (9) et al. indicate that the clinical manifestations of ANK1-HS patients are very similar to those of SPTB-HS, and that the type or location of a single gene mutation cannot be used as a basis for predicting the severity of clinical manifestations (such as anemia, jaundice, etc.) in these patients. Therefore, it is necessary to classify different genetic populations for analysis to guide the development of subsequent treatment plans. Data from Park (16) revealed that SPTA1-HS patients exhibit the most severe clinical symptoms, while SLC4A1-HS patients exhibit the mildest clinical manifestations. In Park's study, although there was no statistically significant difference in disease severity between patients with ANK1 gene mutations and those with SPTB gene mutations, the proportion of children who underwent splenectomy due to SPTB gene mutations (3/31) was significantly lower than that of patients with ANK1 gene mutations (14/44) (*p* = 0.028) (16), indicating that the impact of blood transfusions and splenectomy should be excluded when comparing disease severity between groups. According to the findings of Qin (17) and his team, the mean red blood cell volume and mean hemoglobin content in ANK1-HS patients were higher than those in patients with SPTB mutations. The conflicting conclusions across different regions and populations may stem from the fact that anchor protein 1 (encoded by the ANK1 gene) has three main structural domains: an N-terminal membrane-binding domain, a haptoglobin-binding domain, and a C-terminal regulatory domain (18). Ankyrin is a major protein in the red blood cell membrane, anchoring transmembrane proteins to the cellular membrane skeleton via band 3 protein, haptoglobin, and protein 4.2. Most ANK1 mutations are pathogenic (9, 19–21). Previous studies have shown that patients with hereditary spherocytosis caused by mutations in the spectrin-binding domain exhibit the most severe anemia symptoms compared to those with mutations in other domains (22, 23). Spectrin is a major component of the erythrocyte membrane scaffold network, consisting of α and β subunits, and plays a key role in regulating membrane deformability and mechanical stability (23). The SPTA1 gene encodes α-spectrin, while the SPTB gene encodes β-spectrin, a structural protein of the red blood cell membrane (24). β-Spectrin consists of an N-terminal actin-binding domain and 17 spectrin repeat sequences [including a dimerization domain, partial spectrin repeat sequences, an anchor protein-binding domain, and a tetramerization domain (16)]. Compared to mutations in the SPTA1 gene encoding the α subunit, mutations in the SPTB gene encoding the β subunit are more common. In normal red blood cells, the quantity of α-hemoglobin is three to four times that of β-hemoglobin. Therefore, a mutation in a single β-hemoglobin allele is sufficient to cause HS, while both α-hemoglobin alleles must be mutated to cause the disease (25, 26), Therefore, HS caused by SPTA1 gene mutations accounts for a smaller proportion (5%), and most SPTA1-HS cases only exhibit typical HS-related clinical manifestations when α-hemoglobin expression is reduced to less than 25% of normal levels (27, 28). The data we included suggest that children with ANK1 gene mutation have more severe anemia than those with SPTB gene mutation, which may lead to earlier treatment of children with ANK1 gene mutation and younger age of onset. There is literature (9) showing that HS anemia caused by SLC4A1 gene mutation is mild, and these patients will not see a doctor in childhood, but if it is a study in an adult center, these patients need further analysis, so we did not compare the association between the age of onset of children and the type of gene mutation.The sample size in this study was relatively small, and further expansion of the sample size and collection of patient data prior to blood transfusion and splenectomy are needed for statistical analysis.

This study classified the 35 HS patients included in the study into two major categories based on the type of mutation: nonsense mutations and frameshift mutations, and analyzed the clinical indicators of these two groups. The results showed that the differences between the two groups were not statistically significant (*p* > 0.05), consistent with the findings of Wang D and his team (12). However, due to the relatively small number of HS cases included in this study and the fact that the cases primarily originated from the southwestern region, further research is needed to explore the association between the types of gene mutations and their phenotypes, requiring the inclusion of additional sample resources.

## 7 Summary

At present, there is no clear disease reporting system for hereditary spherocytosis in China, so there is little epidemiological data on HS. This study is the first large-scale clinical retrospective study on HS patients in Southwest China.This study investigated how different types of pathogenic gene mutations affect disease phenotypes and further explored the association between different genotypes of HS and their clinical phenotypes. The study found that children with ANK1-HS had significantly more severe anemia than those with SPTB-HS. Additionally, mutation types did not influence disease severity in this study.

This study has several limitations. First, this is a retrospective study. Many newly diagnosed patients received emergency treatment at local hospitals, and the number of cases treated with blood transfusions may deviate from the actual number. Second, determining baseline hemoglobin levels was challenging when patients were undergoing treatment, as repeated red blood cell transfusions may have been administered during hemolytic crises. This was addressed by calculating the average hemoglobin levels over multiple measurements within 24 months, while excluding samples taken after transfusions. Third, the included patients were young, and the follow-up period was short, making it impossible to assess the necessity and efficacy of splenectomy.

## Data Availability

The original contributions presented in the study are included in the article/Supplementary Material, further inquiries can be directed to the corresponding author.
